# Genetic algorithmic based approach for multiple-objective probabilistic fractional programming problem involving discrete random variables

**DOI:** 10.1371/journal.pone.0274619

**Published:** 2022-09-20

**Authors:** Berhanu Belay, Adane Abebaw

**Affiliations:** Department of Mathematics, Collage of Natural and Computational Sciences, Debre Tabor University, Debre Tabor, Ethiopia; Al-Balqa Applied University Prince Abdullah bin Ghazi Faculty of Information Technology, JORDAN

## Abstract

This manuscript presents a technique for solving a multiple-objective probabilistic fractional programming problem with discrete random variables. A multiple-objective probabilistic mathematical model is constructed with fractional objectives. In the model, some parameters of coefficients and right hand side parameters of restrictions are assumed as random variables having Pascal and Hyper geometric distributions. The feasibility of probabilistic constraints is checked by means of stochastic simulation. Genetic algorithm approach method is used to obtain the Pareto optimal solution of the proposed model without finding the deterministic model. Genetic algorithm parameters are fixed in all generation. The proposed method is coded by C++ programming language. To illustrate the method, a numerical example and practical example on the case of supply chain management are presented. The result shows that the values of the objective functions are conflicting each other.

## Introduction

Fractional mathematical programming is a programming problem where the cost function is the quotient of functions. It is applied when the constraints and cost functions are certain in nature. In other way some real life programming problems have unknown parameters due to uncertain nature. In this case, the uncertainty is handled by using probabilistic programming problem or fuzzy programming problem. A probabilistic fractional programming problems is a mathematical model with some or all data are assumed as random variables.

Optimizing many conflicting fractional objective with respect to the given restrictions is considered as multiple-objective fractional programming (MOFP) problem. When the data of (MOFP) problem are uncertain due to randomness, then it is termed as multiple-objective probabilistic fractional programming (MOPFP) problem.

In multiple-objective programming problem, there exists a set of solutions unlike single objective programming problem, which are superior to the rest of the solutions. The superiority is decided by comparing the values of objective functions. Since multiple-objective programming problems are always in commensurable and conflicting with each other, the best solution is decided by their dominance.

The solution *X*_1_ dominates *X*_2_ if

- In all objective functions *X*_1_ is not worse than *X*_2_.
- At least for one objective function, *X*_1_ is strictly better than *X*_2_.

In other way, non-dominated solution set is the collection of solutions, which are not dominated by other solution set.

Genetic Algorithm (GA) is the heuristic method that works on the concept of survival of the fittest, which is created by John Holland in 1975. The algorithm performs on strings known as individuals of a given population. GA is applied according to four operators. These operators are known as selection (choice), crossover (hybrid), mutation (change), and elitism.

Using a fixed iteration *n*, collection of population *P*(*n*) will be produced. The strings of *P*(*n*) are a solution to the mathematical problem. Utilizing the choice or selection operator, new population is obtained from the old population. The fitter string has a big likelihood of being chosen to the modern population. Then the population takes place the method of hybrid and Change. In hybrid, two parent strings are selected randomly using fixed crossover likelihood (probability) that makes best property of original strings. Using mutation, modern off springs can be made by a few alter within the quality of the chromosome according to mutation likelihood (probability).

After the method of choice, crossover and changes are completed, the other operator, which is called elitism can be connected for advancement of the coming generation. Elitism helps to handle better strings and remove bad strings. There are different elitism operators. Some of these are: alternate, partial and, complete elitism. In this manuscript, complete elitism has been used, which works with the following steps.

1. Merge the population strings before selection and after mutation so that the population is doubled.
2. Sort the resulting population in increasing or decreasing order according to the value of fitness function.
3. Then the first best half strings are selected for next generation. i.e, the strings having good fitness function remain in the system and go to another generation where as the poor strings are discarded.

When we increment the generation, the leading fitted string survives. If the ending criteria reaches, calculation stops and gives the best individual solution for the proposed problem.

The best advantages and practicability of genetic algorithm are:

- GA search the solution from the population of points,not a single point.
- GA uses objective function information,not derivatives
- GA can solve multiple objectives
- GA uses probabilistic orators,not deterministic transitions.
- The concept of GA is easy to understand and use.
- GA is suitable for low computational fitness functions.

Even if GA has the above advantages, it has its own limitation. Some of the limitations are for high computational fitness function or high dimensional problems it takes more computation time(expensive), it needs statistical convergent solution with many simulation, the convergence of the solution depends on initial solution(strings).

We use GA instead of other Heuristic algorithm for multiple-objective cases due to the following reasons:

- the probability of exploration of the search area in GA is very high comparative other heuristic algorithms.
- Simplicity and ease of implementation.
- Large and wide solution space search ability
- Easy to discover global optimum and avoid trapping in local optima.
- The concept of GA is easy to understand and use.
- GA is Parallelism, easily modified and adaptable to different problems
- Genetic representations using chromosomes.

Most real life mathematical programming problems involve more than one fractional objective functions involving uncertain parameters which are conflicting each other. Unlike single objective programming problems, which give unique optimal solution, there may be many non dominated solutions in multiple-objective fractional probabilistic programming problem. Finding the deterministic equivalent of such multiple objective fractional programming under discrete random variable is difficult. As a result, there must be a method that is used to solve such type of problem without finding the deterministic equivalent.

There is lack of studies which solve multiple- objective fractional programming involving discrete random variables using genetic algorithm approach. In addition, the problem has many real life applications. All the above cases, motivate me to conduct and consider the proposed mathematical model as multiple-objective probabilistic fractional programming problem.

This paper has been organized into 6 Sections and at last references do appear. Section 1 focuses on introduction to multiple-objective probabilistic fractional programming and GA. Section 2 presents review of literature. Section 3 is the mathematical model of MOPFP problem. The solution procedure of MOPFP problem is stated in Section 4. Examples for the the proposed model are given in Section 5. At the last section, the conclusion is provided.

## Literature survey

Many authors published the solution methodology for multiple-objective fractional mathematical problems. Such as [1] presented a method by converting into multiple-objective mathematical problem. The challenge of programming problem in real world application is deciding the precise values of available data. The programming problem under randomness is probabilistic model. To get the optimal value of these programming model, it’s deterministic equivalent is found by taking the data as continuous random variable [2, 3]. Some other researchers [4–7] proposed a method for stochastic programming problems having multiple-objectives. But, getting the deterministic of probabilistic model is time wastage and even difficult. To overcome this difficulty different researchers suggested GA for mathematical problem involving continuous random variable [8–11]. If few of the data of fractional mathematical problems are described by random variable, it is referred to as stochastic fractional programming. Zhu and Huang [12] stated the use of fractional mathematical problem involving random variables in the use of electric power. Das and Mandel [13] solved stochastic fractional programming problems by transforming to deterministic fractional problem. [14] showed the application of probabilistic fractional goal programming involving multiple-objectives for the distribution of water resource in industries. GA is proposed to find chance constraint fractional programming by handling the probabilistic limitations using stochastic simulation [15–17]. In some real life circumstances, all or some data of the probabilistic programming problem are expressed by discrete random variables. Though, the solution method of MOPFP problem involving discrete distribution does not appear in the literature. In stochastic programming problem where it is difficult to find its deterministic, [9] have estimated the probabilistic constraints by using random variable generation and stochastic simulation. Different researchers solved single objective probabilistic programming having random variables with discrete distributions [18–20].

From the literature that we observed, many authors tried to solve multiple-objective probabilistic programming problem by finding the deterministic equivalent. In addition some authors solved multiple-objective fractional programming problems by using classical method of fractional programming by transforming into equivalent multiple-objective programming problem. All these methods take time and even difficult to find the deterministic equivalence when the parameters involve some discrete random variables like hyper geometric and pascal distributions.

Recently, [21] presented an optimization model for software quality prediction with case study analysis using MATLA. [22] proposed Consensus based combining method for classifier ensembles. [23] studied efficient malware detection approach with feature weighting based on Harris Hawks optimization. [24] Eccentric methodology with optimization to unearth hidden facts of search engine result. [25] studied a new insight on solving fuzzy linear fractional programming in material aspects. [26] proposed fuzzy nonlinear programming approach for multi-objective sum of linear and linear fractional programming problem. [27] proposed a numerical approach for solving linear fractional programming problem in a fuzzy environment. [5] presented multi-objective probabilistic fractional programming problem involving two parameters Cauchy distribution. [28] Studied on probabilistic multi-objective linear fractional programming problems under fuzziness. [29] proposed elite artificial bees’ colony algorithm to solve robot’s fuzzy constrained routing problem [30] studied application of multi-objective probabilistic fractional programming problem in production planning. [31] studied application of fuzzy random-based multi-objective linear fractional programming to inventory management problem.

So in this manuscript, we have made an attempt for solving MOPFP problem having discreet random variables using stochastic simulation based genetic algorithm.

## Formulation of MOPFP problem

MOPFP Problem is expressed as:

$$\begin{array}{c} \text{max}:Z_{q}=\frac{N_{q}\left(x_{j}\right)}{D_{q}\left(x_{j}\right)}q=1,2,\ldots Q \end{array}$$ (1)

subject to

$$\begin{array}{c} P\left(\sum_{j=1}^{n}a_{ij}x_{j}\leq b_{i}\right)\geq \alpha_{i},i=1,2,\ldots ,m \end{array}$$ (2)

$$\begin{array}{c} 0<\alpha_{i}<1,i=1,2,\ldots ,m \end{array}$$ (3)

$$\begin{array}{c} x_{j}\geq 0,j=1,2,\ldots ,n \end{array}$$ (4)

where *N*_*q*_(*x*_*j*_) and *D*_*q*_(*x*_*j*_) are function of *x*_*j*_. “P” is the probability, *α*_*i*_ is the number in between 0 and 1 which is the violation.

In this manuscript, we consider the parameter in the coefficient of constraint and the right hand side are hyper-geometric and Pascal random variables respectively.

Suppose that *x* is a random variable:

(*a*): *x* has Pascal distribution with known parameters *n* and *p*, whose probability mass function is equal to:

$$\begin{array}{c} f\left(x\right)=\{\begin{array}{cc} \frac{(x-1)!}{(x-n)!(n-1)!}p^{n}{\left(1-p\right)}^{x-n}, & \text{if}\;x=n,n+1,n+2,\ldots ,(0<p<1)\\ 0, & \text{otherwise} \end{array} \end{array}$$

it is denoted by *PS*(*n*, *p*)

(*b*): *x* has Hyper geometric distribution, if the probability mass function is expressed:

$$\begin{array}{c} f(x)=\frac{\left(\begin{array}{c} n_{1}\\ x \end{array})(\begin{array}{c} N-n_{1}\\ m-x \end{array}\right)}{\left(\begin{array}{c} N\\ m \end{array}\right)},\text{max}(0,n_{1}+m-N)\leq x\leq \text{min}(n_{1},m) \end{array}$$

and it is denoted by *H*(*N*, *m*, *n*1). It is a discrete distribution of sampling without replacement. The parameters *N*, *m*, *n*_1_, *N* − *n*_1_ represents total population size in both classes, sample size, first class population size and second class population size respectively.

## Solution procedure

The compromise solution of the proposed mathematical programming is obtained by stochastic simulation together with GA. In this case:

- Finding deterministic equivalence of chance constraint programming problem is not required
- Transforming MOFP problem into non fractional mathematical problem is also not required.

The algorithms is described by the following procedure:

*step 0*: Specify the parameters such as crossover probability (pc), mutation probability (pm), number of generation (*t*) and distribution parameters.
*step 1*: Randomly initialize the population *P*(*t*) of GA.
*step 2*: Based on the choice making circumstance, distinguish the two bounds the chosen variables.
*step 3*: Calculate the functions to be optimized.
*step 4*: Test the likelihood condition of the limitations. In the event that the condition is satisfied at that point go to following step else go to step 1.
*step 5*: Apply selection operator to select the best chromosomes from the population.
*step 6*: Apply crossover operator to produce new pair of chromosomes.
*step 7*: Mutation operation is applied to the existing population of chromosomes to have variety in the present population.
*step 8*: Evaluate the functions to be optimized again.
*step 9*: Check the likelihood criteria of the limitations once more. In the event that the criteria is fulfilled at that point go to following step else go to step 1.
*step 10*: Apply Elitism to the present population.
*step 11*: Check whether max number of generation is achieved or not, if not go to step-3.
*step 12*: When the end condition is come to the show, the present populace gives the leading solution.

The pseudo-code for stochastic simulation based GA is described by:

*Begin*

*t = 1*;

*initialize population P(t)*;

*calculate fitness function*;

*while(termination criteria is not reached)*

*do*

*t = t+1*;

*check probability criteria*;

*Choose P(t) from p(t-1)*;

*applying crossover*;

*applying mutation*;

*evaluate fitness function p(t+1)*;

*check probability criteria*;

*apply elitism*;

*end while*

*end begin*

**Fig 1:**
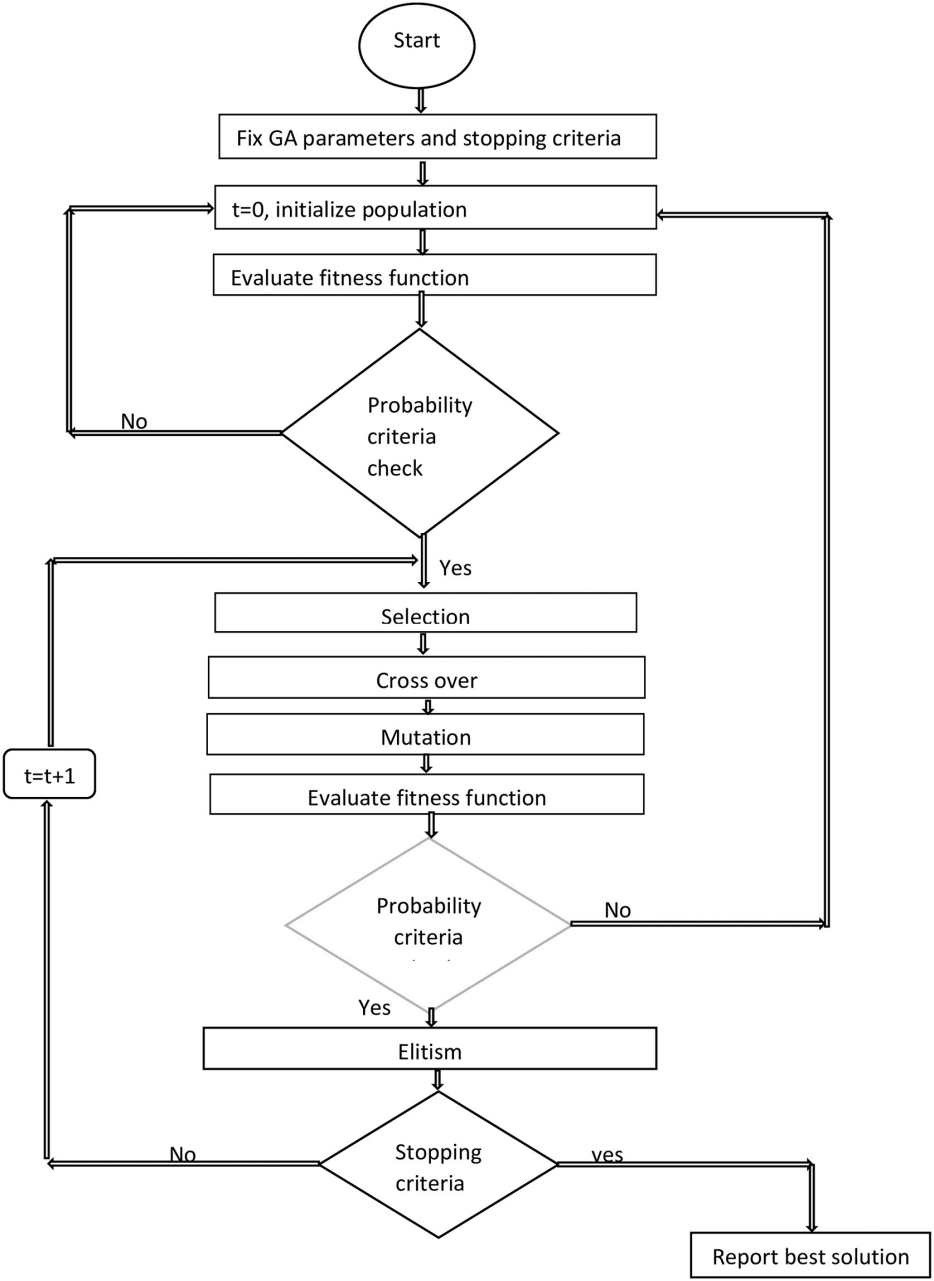
Stream chart of GA.

The stream chart for stochastic simulation together with GA is described by Fig 1.

## Examples

One numerical example and one real life application will be discussed here as follows.

### Numerical example

Consider the multiple-objective probabilistic fractional programming.

$$\begin{array}{c} \text{max}:Z_{1}=\frac{x_{1}+6x_{2}-3x_{3}}{3x_{1}+8x_{2}+x_{3}+4} \end{array}$$

$$\begin{array}{c} \text{max}:Z_{2}=\frac{-6x_{1}+7x_{2}+2x_{3}}{2x_{1}+3x_{2}+6} \end{array}$$

subject to

$$\begin{array}{c} P(a_{1}x_{1}-4x_{2}-a_{2}x_{3}\leq b_{1})\geq 0.75 \end{array}$$

$$\begin{array}{c} P(5x_{1}-a_{3}x_{2}-a_{4}x_{3}\leq b_{2})\geq 0.55 \end{array}$$

$$\begin{array}{c} P(a_{5}x_{1}-a_{6}x_{2}+3x_{3}\leq b_{3})\geq 0.65 \end{array}$$

$$\begin{array}{c} 0\leq x_{i}\leq 5,i=1,2,3. \end{array}$$

where (*i*): *a*_1_, *a*_2_, *a*_3_, *a*_4_, *a*_5_, *a*_6_ are Pascal distribution with two parameters *n* and *p*: 4 and 0.6, 5 and 0.7, 3 and 0.8, 5 and 0.9, 2 and 0.75, 3 and 0.85 respectively.

(*ii*): *b*_1_, *b*_2_, *b*_3_ are hyper-geometric random variables with three parameters *N*, *m*, *n*_1_: 40, 17, 15 and 10, 8, 4 and 50,24,20 respectively.

To discover the Pareto ideal solutions, we utilize stochastic recreation based GA. The stochastic reenactment GA is coded by utilizing C++ programming dialect. Within the calculation, we utilize competition determination, one point cross over and bit wise transformation administrators and the greatest generation as stopping criteria. The other parameter are given as: size of population *p*(*t*) = 100, *p*_*m*_ = 0.001 and *p*_*c*_ = 0.7, and maximum generation *t* taken as 200.

In this manuscript the GA parameters are fixed in all cases. The population size should not be very large as a huge computational time is required and which causes GA to slow down, while smaller population might not be enough for a good mating pool. Therefore, an optimal population size needs to be decided by trial and error. That is why we choose the population size *p*(*t*) = 100. Crossover has a higher probability, typically 0.7–0.95 is recommended. The experiments we did with crossover showed that a rate of 80% is good enough. This means that *pc* = 0.7 is sufficient for our problem. On the other hand, mutation is carried out by flipping some digits of a string, which generates new solutions. For our problem, Mutation rate is in the range of [0.001, 0.05]. In general, this mutation probability is typically low, from 0.001 to 0.05 is recommended for GA. In our cases *pm* = 0.001 is good choice. Number of generation refers to the number of cycles before termination. In some cases 100 loops are sufficient, but in the other cases more generation is needed. This depends on the problem type and its complexity. So in our problem *t* = 200 generation is sufficient.

**Fig 2:**
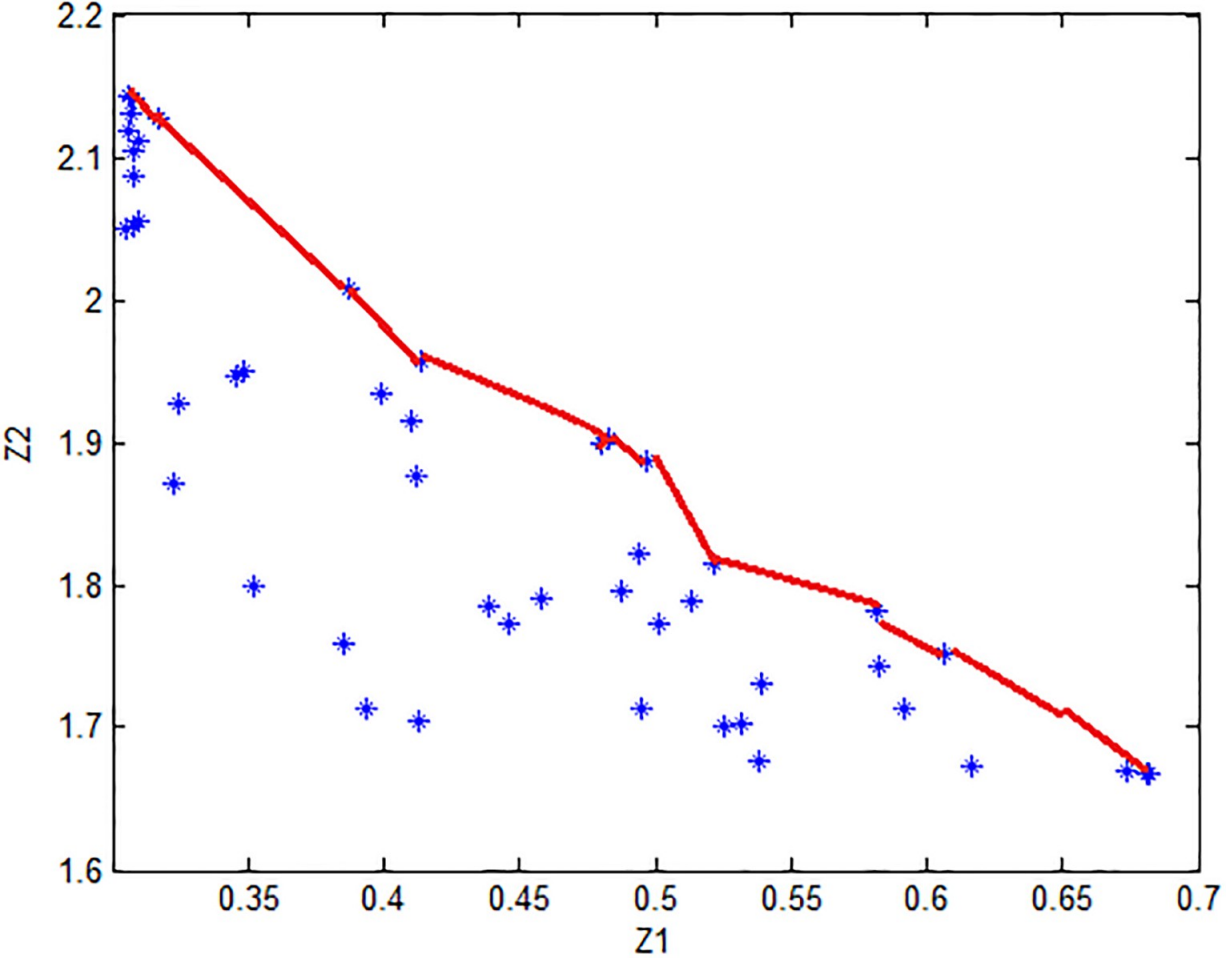
Set of non dominated points.

Solving the problem using stochastic simulation GA, we got non dominated solutions which is given by Table 1 with 200 generations. The set of non dominated points are expressed by Fig 2.

**Table 1:** Non dominated solutions and values of *Z*_1_ and *Z*_2_.

*x*_1_	*x*_2_	*x*_3_	*Z*_1_	*Z*_2_
0	4.98045	0.909091	0.606789	1.75162
0	4.98045	1.22678	0.581365	1.78196
0	4.99511	4.73118	0.32299	2.11665
0	4.98045	2.31672	0.4968027	1.88606
0	5	2.45357	0.487353	1.90034
0	4.9609	4.15934	0.361309	2.06127
0	5	0.410557	0.646534	1.70452
0	5	2.70772	0.468377	1.92454
0	5	2.12121	0.512484	1.86869
0	5	0.0293255	0.679366	1.66946
0	5	2.51222	0.482956	1.90593
0	5	3.75367	0.39241	2.02416
0	5	22.6784	0.470556	1.92175
0	5	4.3695	0.349218	2.08281

As we see the results,the non dominated or compromise solutions are conflicting each other. This means that when *Z*_1_ is best then *Z*_2_ is worst and vice versa. In this case the decision maker can choose one of the best solution depending on his/her interest.

The uncertain coefficients of the constraints are approximated by stochastic simulation based GA method. The method generates infinity many solutions,among these solution the 14 solutions are none dominated solutions.

### Real life example of supplier selection in supply chain problem

Suppliers have a great role in manufacture or purchasing to satisfy the need of the customers. The main strategy of the decision maker in supply chain management is selecting the best suppliers that archive the targeted goal. In practical supplier selection is affected by conflicting criteria. The most important criteria are quality, cost and delivery time. In addition to this some parameters like total demand and maximum capacity of suppliers are affected by uncertainty. This uncertainty can be characterized by random variables having known distributions. Hence probability theory is the best tool to handle uncertainty. In this example, we develop a multi-objective fractional programming problem for supplier selection problem subject to probabilistic constraints with uncertain demand and maximum capacity of suppliers.

Suppose that 4 suppliers are needed for supplying new products to a market. The market manager designed criteria for selecting suppliers based on cost of product, quality of product and delivery time. It is assumed that the total demand of the market and maximum capacity of each supplier are random variables following Pascal distribution and hyper-geometric distribution. The manager wants to select suppliers which minimize cost per late time deliver ratio and maximize quality of product per on time delivery ratio subject to probabilistic demand and supplier capacity. The supplier quantities are given by Table 2.

**Table 2:** Supplier quantity data.

supplier	*c*_*i*_	*q*_*i*_	*o*_*i*_	*l*_*i*_	*NC*_*i*_	*mC*_*i*_	*n*_1_ *C*_*i*_
1	4	75	65	35	100	75	50
2	2	80	75	25	80	60	40
3	3	95	80	20	90	65	50
4	5	65	90	10	120	80	65

#### Model development

The supplier selection model is multiple-objective fractional programming problem with probabilistic constraints.

The cost functions are:

1. the quality of item per on time delivery.i.e
2. cost per late time delivery

the cost functions are evaluated under the following limitations.

1. the total product supplied by suppliers is not less than the total demand of the costumer.
2. the capacity of each supplier can not exceed from the the maximum capacity.

Therefore, the general mathematical model of suppliers selection is expressed by:

$$\begin{array}{c} max:Z_{1}=\frac{\sum_{i=1}^{4}q_{i}x_{i}}{\sum_{i=1}^{4}o_{i}x_{i}} \end{array}$$ (5)

$$\begin{array}{c} min:Z_{2}=\frac{\sum_{i=1}^{4}c_{i}x_{i}}{\sum_{i=1}^{4}l_{i}x_{i}} \end{array}$$ (6)

subject to

$$\begin{array}{c} P(\sum_{i=1}^{4}x_{i}\geq D)\geq \beta \end{array}$$ (7)

$$\begin{array}{c} P\left(x_{i}\geq C_{i}\right)\geq \alpha_{i} \end{array}$$ (8)

$$\begin{array}{c} x_{i}\geq 0.i=1,2,3,4. \end{array}$$ (9)

where: *q*_*i*_: percentage of quality of product of supplier *i*.

*o*_*i*_: percentage of on time delivery of supplier *i*

*l*_*i*_: percentage of late time delivery of supplier *i*

*c*_*i*_: cost of product supplied by supplier *i*

*D*: uncertain demand of the costumer which follows Paskal distribution with two parameters *n* and *p*

*C*_*i*_: uncertain maximum capacity of supplier *i* which follow beta distribution with three parameters *N*, *m*, *n*_1_.

*x*_*i*_: quantity of supplier *i*.

*P*: is the probability

*α*_*i*_ and *β* are the given probabilities at which the constraint violations are admitted.

Substituting all the data values given in Table 2, we obtain the following multiple-objective fractional probabilistic programming problem:

$$\begin{array}{c} max:Z_{1}=\frac{0.75x_{1}+0.80x_{2}+0.95x_{3}+0.65x_{4}}{0.65x_{1}+0.75x_{2}+0.80x_{3}+0.9x_{4}} \end{array}$$ (10)

$$\begin{array}{c} min:Z_{2}=\frac{4x_{1}+2x_{2}+3x_{3}+5x_{4}}{0.35x_{1}+0.25x_{2}+0.2x_{3}+0.1x_{4}} \end{array}$$ (11)

subject to

$$\begin{array}{c} P(x_{1}+x_{2}+x_{3}+x_{4}\geq D)\geq 0.7 \end{array}$$ (12)

$$\begin{array}{c} P(x_{1}\geq C_{1})\geq 0.65 \end{array}$$ (13)

$$\begin{array}{c} P(x_{2}\geq C_{2})\geq 0.85 \end{array}$$ (14)

$$\begin{array}{c} P(x_{3}\geq C_{3})\geq 0.45 \end{array}$$ (15)

$$\begin{array}{c} P(x_{4}\geq C_{4})\geq 0.35 \end{array}$$ (16)

$$\begin{array}{c} x_{1},x_{2},x_{3},x_{4}\geq 0. \end{array}$$ (17)

where (*i*): *D* is random variable following Pascal distribution with two parameters *n* = 5 and *p* = 0.01:

(*ii*): *C*_1_, *C*_2_, *C*_3_, *C*_4_ are random variables following hyper-geometric distribution with three parameters *N*, *m*, *n*_1_ having value given in Table 2.

#### Results and discussion

The non dominated solutions are gotten utilizing stochastic recreation based GA by utilizing *p*(*t*) = 100, *p*_*c*_ = 0.7, *p*_*m*_ = 0.001 and *t* = 200 and dissemination data stated above. Coding stochastic reenactment based GA utilizing C++, we get the taking after non dominated ideal solutions of supplier determination demonstrate. Table 3 appears the dominated solutions and ideal values of the two objective functions.

**Table 3:** Non dominated solutions and values of *Z*_1_ and *Z*_2_.

*x*_1_	*x*_2_	*x*_3_	*x*_4_	*Z*_1_	*Z*_2_
1.17302	14.9756	9.83382	0	1.11788	10.5805
3.75367	19.9609	12.4927	0	1.11849	10.7273
2.73705	17.4976	9.52102	0	1.11442	10.4943
1.54448	14.4477	7.46823	0	1.1121	10.3212
0.938416	17.6344	9.05181	0	1.1107	10.1805
2.24829	10.7918	8.79765	0	1.1256	11.101
16.9697	13.001	7.46823	0	1.12959	11.8239
16.6569	19.7263	19.824	0.0391007	1.13527	11.9266
16.2659	18.7488	20	0	1.13693	11.9821
0	20	20	0	1.12903	11.1111
9.97067	8.11339	12.45367	0.0195503	1.4485	12.4554
14.1349	5.8651	19.2571	0	1.1585	13.1867
16.0899	7.46823	19.5112	0	1.15501	13.0074
1.19257	5.45455	19.5699	0	1.162141	13.2003
0.449658	4.69208	15.5425	0	1.16072	13.0899
1.75953	3.57771	16.0117	0	1.1657	13.4557
8.97361	3.42131	9.16911	0	1.15532	13.0536

**Fig 3:**
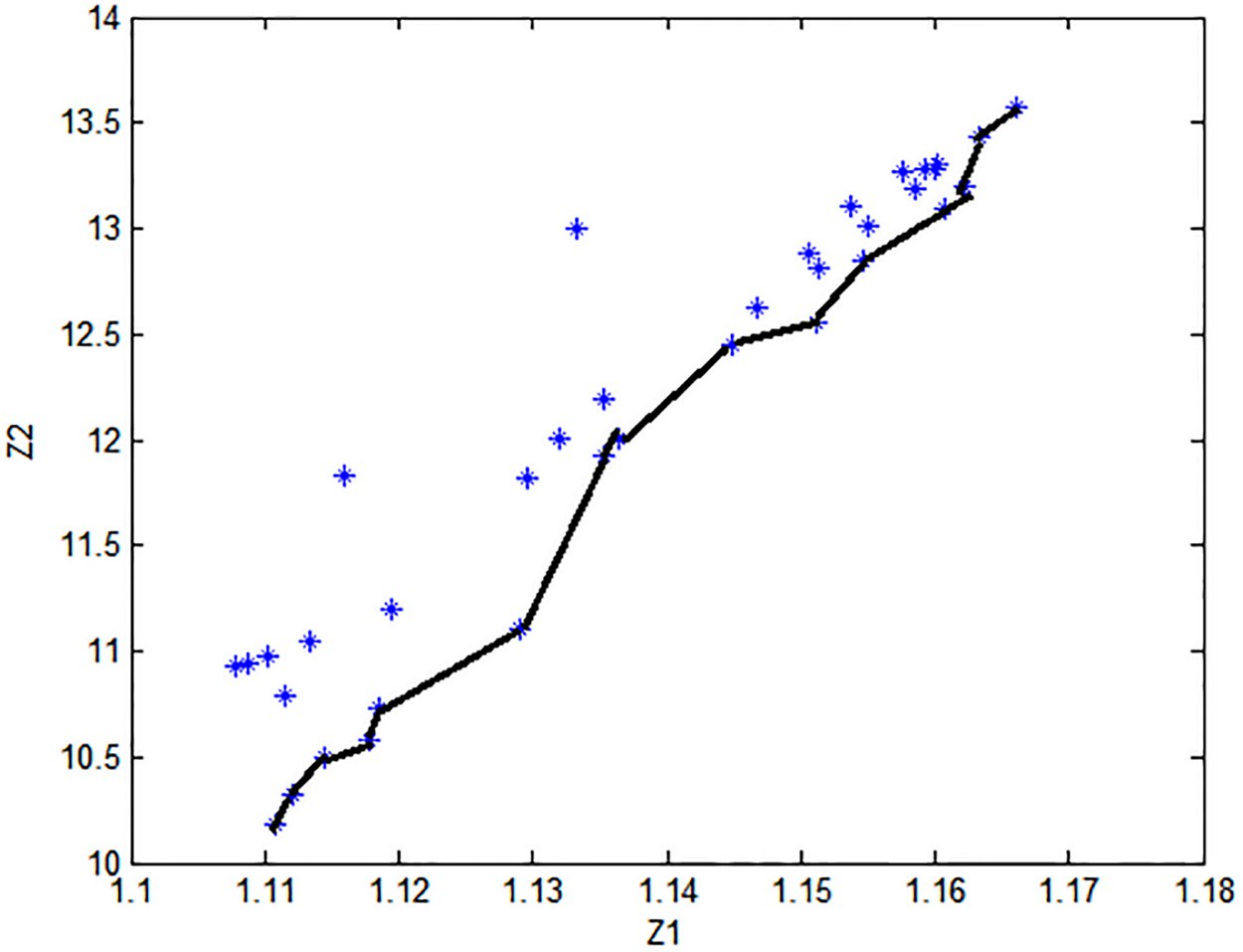
Non dominated solution.

The pareto frontier points the supplier selection problem are expressed by Fig 3.

## Conclusion

In this manuscript, MOPFP problem has been solved using stochastic simulation together with GA by waving the deterministic identical of the problem. The method doesn’t require any classical method of fractional programming to convert the multiple-objective fractional programming problem into identical multiple-objective programming problem. The method is simple and easy to use which is coded by C++ programming language. Moreover, GA helps to generate many solutions at a time which are non dominated solutions. This is important for decision makers to make a good decision by considering all the possible directions. In the proposed method, when the number of iteration increases the probability of getting best non dominated solution is very high.

Since most real life problems ar uncertain due to randomness, stochastic simulation can easily handle the probabilistic constraints. For such type of mathematical problem the proposed method is used to get the non dominated solution for multiple-objective fractional uncertain problems for which the decision maker can select best supplier among many suppliers.

We recommend that the proposed problem can be solved by other heuristic algorithms like particle swarm algorithm and ant colony algorithm. In addition the problem can extended to fully fuzzy multi-objective probabilistic fractional programming problem and multi-level multi objective probabilistic fractional programming problem that involves both discrete and continuous random variables.
